# Editorial: mHealth: Self-Management and Complementary Psychiatric Treatment

**DOI:** 10.3389/fpsyt.2021.777090

**Published:** 2021-10-22

**Authors:** Eric D. Achtyes, Lina Gega, Outi Linnaranta

**Affiliations:** ^1^Pine Rest Christian Mental Health Services, Grand Rapids, MI, United States; ^2^Division of Psychiatry and Behavioral Medicine, Michigan State University College of Human Medicine, Grand Rapids, MI, United States; ^3^Department of Health Sciences and Hull York Medical School, University of York, York, United Kingdom; ^4^Mental Health Team, Finnish Institute for Health and Welfare, Helsinki, Finland; ^5^Faculty of Medicine, Department of Psychiatry, McGill University, Quebec City, QC, Canada

**Keywords:** digital mental health, telepsychiatry, internet, mobile applications, virtual reality, severe mental illness, relapse prevention

The need to disseminate medical and psychological interventions using technology has been a pressing issue for psychiatric populations who are significantly underserved by traditional health services. As a case in point, only 44.8% of adults with mental illness in the United States have received treatment in 2019 (1). mHealth refers to services and devices, such as online programmes, mobile applications, virtual reality (VR) systems and videogames, that can be used to identify and support those with mental health needs beyond the traditional one-to-one conversations in a clinic with a doctor or therapist (2–4).

The purpose of this special issue was to capture recent advances in the way technology—under the umbrella of mHealth—can be used to understand and monitor the manifestation and trajectory of emotional and psychiatric problems. The included papers illustrate how mHealth can be used to support treatment and care, from fully automated self-management and monitoring systems to blended interventions in which technology-enabled self-care is supported by a clinician, or clinician-led care is supplemented by technology.

Three articles in this collection relate to schizophrenia and psychotic disorders. The first paper by Buck et al. suggests that frequent measurements delivered by mobile technologies could be leveraged to detect increases in psychotic symptoms that may precede relapses. Gowarty et al. found that two smoking cessation mobile apps were usable and appealing among young adult smokers with psychotic disorders; however, coaching at the start of treatment and app notifications throughout treatment were important to promote uptake and engagement. Finally, Hänsel et al. identified less color, lower saturation, and fewer faces in uploaded images on Instagram by individuals with schizophrenia compared to healthy volunteers, suggesting that image-sharing social media platforms can be an intriguing medium for prediction, identification, and monitoring of serious mental illness.

Two studies were conducted at the interface of physical and mental health. First, Duthely et al. designed a mobile two-way texting system that sends and receives simple messages to promote adherence to anti-retroviral medications and medical appointments for ethnic minority women living with HIV/AIDS. The authors concluded that depression was a significant predictor of viral non-suppression and that mHealth has the potential to supplement medical care in this population, once patient concerns about privacy and confidentiality are addressed. The second study by Janney et al. identified patient preferences for three different wearables to promote physical activity in bipolar disorder. The armband (worn around the upper arm) was the favorite, followed by the pedometer (worn around the hip), and the actigraph (worn around the waist). Interestingly, the order of preference was the reverse to the order of accuracy of activity monitoring and reported problems (e.g., skin irritation), and may have been influenced by factors such as being able to wear the device discreetly or conceal it under clothes. Both studies flagged patient preferences that could influence uptake and usage of mHealth devices.

Virtual reality featured in two papers that discussed mechanisms of reducing stress and fear. The first paper by Lindner et al. used a VR system to enable graded, controlled and repeated exposure to phobic stimuli associated with spiders. Through longitudinal modeling that indirectly compared two studies, one with instructions for real-life exposure virtual reality post-therapy and one without such instructions, the paper suggests that virtual exposure paired with real-life exposure can maximize fear reduction as part of therapy. The second paper by Kim et al. used VR natural scenes with a soothing soundtrack to induce relaxation in individuals with high stress, as an alternative to traditional biofeedback. The two relaxation methods showed no differences in total perceived stress, but differential effects on physiological outcomes suggested that VR may be more effective for relaxation by means of parasympathetic activity and traditional biofeedback by reducing muscle tension.

Finally, a brief report by Grunebaum et al. is the first to show evidence of increased assay sensitivity of computerized adaptive testing over traditional measures in a clinical trial with participants who experienced suicidal depression. The importance of this work is in showing that brief phone or computer-based long-term follow-up assessments can be a reliable method in detecting suicide risk and has the added value of minimizing repeated measurement bias that can occur with traditional fixed-length assessments.

This collection of eight articles exemplifies the diverse ways in which mHealth can be applied across mental health conditions and vulnerable populations, including suicidal depression, generalized anxiety, specific phobia, HIV/AIDS, bipolar disorder, schizophrenia, and psychotic disorders. With reference to wearable technologies, a computerized adaptive test, a mobile assessment system, two VR systems, a text messaging service, Instagram and mobile apps, these eight articles demonstrate how mHealth can support physical activity, smoking cessation, psychiatric treatment adherence, detection of onset or relapse of mental illness, symptom severity monitoring, stress management, and psychological therapy.

One caveat that permeates all papers in this special issue is that mHealth remains largely experimental and confined within research settings, rather than being deployed in the real world and in routine clinical settings. Digital media can overcome geographical barriers and make the most of the available workforce through remote consultations, task-shifting and self-management; however, large-scale implementation of digital psychiatry is slow and limited (5). A silver lining of the COVID-19 pandemic is the growing use and acceptability of technology as means to delivering psychiatric care, which can be a steppingstone to scale up the provision of care post-pandemic to those who need it the most (6, 7).

Most mHealth evidence to-date relates to brief self-help interventions for anxiety disorders and depression of mild-to-moderate severity in the general population (8). The contribution of the collection of articles in this special issue is, first, in demonstrating mHealth's potential to assess and support people with a range of severe psychiatric conditions, such as schizophrenia, bipolar disorder and suicidal depression, and second, in using mHealth in creative and diverse ways, from reducing vulnerability to stress before clinical symptoms develop, to preventing relapse for those who have a clinical diagnosis and receive psychiatric treatment.

## Author Contributions

All authors contributed to the design, review and editing of the Research Topic, and to the Editorial summarizing it.

## Conflict of Interest

The authors declare that the research was conducted in the absence of any commercial or financial relationships that could be construed as a potential conflict of interest.

## Publisher's Note

All claims expressed in this article are solely those of the authors and do not necessarily represent those of their affiliated organizations, or those of the publisher, the editors and the reviewers. Any product that may be evaluated in this article, or claim that may be made by its manufacturer, is not guaranteed or endorsed by the publisher.
